# Teratogenic and Embryocidal Effects of Zidovudine (AZT) in Sprague-Dawley Rats

**DOI:** 10.1155/S1064744995000068

**Published:** 1995

**Authors:** James T. Christmas, Bertis B. Little, Kraig A. Knoll, Roger E. Bawdon, Larry C. Gilstrap III

**Affiliations:** Division of Prenatal Diagnosis Department of Obstetrics & Gynecology University of Texas Southwestern Medical Center 5323 Harry Hines Boulevard Dallas TX 75235-9032 USA

## Abstract

*Objective:* The purpose of the present investigation was to analyze the effets of zidovudine on the postimplantation embryo and fetus.

*Methods:* Pregnant Sprague-Dawley rats were given various doses (10 mg/kg, 30 mg/kg, 150 mg/kg) of zidovudine or saline by an endotracheal tube during the period of embryogenesis (days 6–8, 9–11, 6–11 postconception). The animals were sacrificed on days 18–19 of pregnancy, and their fetuses were removed by hysterotomy. Autopsies under low (15×) and high (40×) power light microscopy were performed on all fetuses.

*Results:* There was no statistically significant difference among the groups with respect to maternal weight gain. There were more pregnancy resorptions in the group receiving high-dose zidovudine (150 mg/kg/day) throughout embryogenesis than in the control group (*P* = 0.001, respectively). Four major structural anomalies were noted among the 689 fetuses examined, but zidovudine was not associated with an increased frequency of congenital anomalies in rats when it was administered in doses similar to, 3-, and 15-fold higher than the regimen recommended for adult humans. The drug, however, was embryocidal in the high-dose group (*P* = 0.002).

*Conclusions:* These findings are consistent with previous studies of preimplantation mouse embryos that demonstrated an embryocidal effect on preimplantation conceptuses. In summary, post-implantation embryonic zidovudine exposure was associated with significantly increased pregnancy losses (resorptions and intrauterine deaths).


Infectious Diseases in Obstetrics and Gynecology 2:223–227 (1995)
© 1995 Wiley-Liss, Inc.

### Teratogenic and Embryocidal Effects of Zidovudine (AZT) in Sprague-Dawley Rats

James T. Christmas, Bertis B. Little, Kraig A. Knoll, Roger E. Bawdon, and Larry C. Gilstrap III

*Department of Obstetrics & Gynecology, University of Texas Southwestern Medical Center, Dallas, TX*

ABSTRACT

***Objective:*** **The purpose of the present investigation was to analyze the effets of zidovudine on the postimplantation embryo and fetus.**

***Methods:*** **Pregnant Sprague-Dawley rats were given various doses (10 mg/kg, 30 mg/kg, 150 mg/kg) of zidovudine or saline by an endotracheal tube during the period of embryogenesis (days 6–8, 9–11, 6–11 postconception). The animals were sacrificed on days 18–19 of pregnancy, and their fetuses were removed by hysterotomy. Autopsies under low (15×) and high (40×) power light microscopy were performed on all fetuses.**

***Results:*** **There was no statistically significant difference among the groups with respect to maternal weight gain. There were more pregnancy resorptions in the group receiving high-dose zidovudine (150 mg/kg/day) throughout embryogenesis than in the control group ($P$ = 0.001, respectively). Four major structural anomalies were noted among the 689 fetuses examined, but zidovudine was not associated with an increased frequency of congenital anomalies in rats when it was administered in doses similar to, 3-, and 15-fold higher than the regimen recommended for adult humans. The drug, however, was embryocidal in the high-dose group ($P$ = 0.002).**

***Conclusions:*** **These findings are consistent with previous studies of preimplantation mouse embryos that demonstrated an embryocidal effect on preimplantation conceptuses. In summary, postimplantation embryonic zidovudine exposure was associated with significantly increased pregnancy losses (resorptions and intrauterine deaths).** © 1995 Wiley-Liss, Inc.

KEY WORDS

Postimplantation embryos, congenital anomalies, resorption, intrauterine death

Zidovudine, formerly called azidothymidine (AZT), was the first agent commercially available for the treatment of symptomatic HIV infection and was shown to be effective.[1–3] The current recommendations from the Centers for Disease Control (CDC) are that zidovudine therapy be offered to pregnant patients with HIV and near normal CD4 counts to prevent perinatal transmission of HIV.[4]

AIDS was previously considered a disease primarily of homosexual men,[5] but recent seroprevalence studies indicate that the male-female ratio may be much closer to 1:1. Certain inner city obstetric populations have been shown to have seroprevalence rates as high as 2%, and nearly 50% of the sexual partners of HIV-infected IV drug abusers demonstrate serologic evidence of infection.[6,7] Recent reports indicate that the prevalence of HIV approaches 1 in 250 in the general population, but the rate among pregnant women is much lower, at 1–2 in 1,000.

Zidovudine acts as an antiviral agent by inhibiting viral reverse transcriptase and by terminating viral DNA chain elongation.[8] Host cellular DNA polymerase is also inhibited, but only at concentrations 100 times greater than those required to inhibit viral reverse transcriptase in mammals.[9] The side effects commonly encountered in therapeutic

**Address correspondence/reprint requests to Dr. Bertis B. Little, Division of Prenatal Diagnosis, Department of Obstetrics & Gynecology, University of Texas Southwestern Medical Center, 5323 Harry Hines Boulevard, Dallas, TX 75235-9032.**

Received August 22, 1994
Accepted November 15, 1994
Basic Science Article trials suggest that in vivo this agent may not be entirely virus-specific. In one study, 24% of zidovudine recipients developed anemia (hemoglobin ≤7.5 g/dl), and 16% developed neutropenia (<500 cells/$mm^3$).[10]

Animal studies have also shown rapid placental transfer of zidovudine,[12] and zidovudine has been shown to cross the placenta in explanted human placental cotyledon studies.[13,14] The drug may disrupt early embryonic development because, as mentioned previously, the mechanism of action of zidovudine is the inhibition of DNA replication. Zidovudine was associated with increased preimplantation mouse embryo loss in several studies,[15–18] but no congenital abnormalities were detectable. The possible mechanism of embryolethality of zidovudine was thought to be due to retarded cell division.[18] Zidovudine is classified by its manufacturer as a pregnancy Category C drug. The manufacturer quotes a single unpublished study in pregnant rats using doses up to 20 times the human dose that revealed "no evidence of harm to the fetus due to zidovudine."[11] Its use in pregnancy is recommended only if the potential benefits for the mother justify the potential risks to the embryo or fetus.

Previous investigations suggested that congenital anomalies may be an effect of exposure to zidovudine during organogenesis. The purpose of the present study was to test this hypothesis by administering zidovudine to pregnant rats during organogenesis.

### SUBJECTS AND METHODS

This study was approved by the Institutional Review Board for Animal Research of the University of Texas Southwestern Medical Center. Virgin female Sprague-Dawley rats were obtained from a commercial vendor and allowed to acclimate for 7 days before mating, at which time the females were placed with males overnight for mating. Vaginal smears were examined daily and the presence of spermatozoa on microscopic examination was considered evidence of pregnancy. The date of the positive smear was recorded as day zero of pregnancy.

The contents of zidovudine capsules were suspended in a sterile water solution with a pH of 8.0. All animals were dosed by intragastric intubation. The concentration of zidovudine in the solution was validated by high-pressure liquid chromatography in our laboratory using laboratory-assay zidovudine powder provided by the manufacturer (Burroughs Wellcome, Research Triangle Park, NC) as a standard. All animals were maintained at the Animal Resource Center of the University of Texas Southwestern Medical Center for the duration of their pregnancies. They had a supply of regular rat chow and water available ad libitum. The experimental animals were weighed daily but otherwise had no handling except for the administration of medication or placebo. The daily doses were adjusted according to the daily weights.

Ninety pregnant rats were assigned at random to one of five groups with 18 per group. The animals in group I received zidovudine, 10mg/kg/day, in a single oral morning dose on days 6–11 of pregnancy. Those in group II and group III each received zidovudine, 30 mg/kg/day, in a single oral morning dose on days 6–8 and 9–11, respectively; the pregnant animals in group IV received 150 mg/kg/day of zidovudine orally on days 6–11 postconception. The initial experimental design was to subdivide the high-dose group into days 6–8 and 9–11. The high-dose group was not subdivided into early (days 6–8) and late (days 9–11) because no congenital anomalies were noted in the high-dose group treated throughout the period of embryogenesis. The animals in group V served as controls and were given an equal volume of sterile water as a single oral morning dose by intragastric intubation on days 6–11 of pregnancy. This number of animals was chosen to comply with the published recommendations for the design of subprimate animal studies.[19] The absorption of zidovudine through intragastric intubation was documented in a prior investigation conducted in this laboratory in which the placental transfer was analyzed.[12]

The greatest number of anatomic structures form in the rat embryo in the period of gestation from days 6–11 (taking insemination as day 0). By day 11, the neural structures have formed, somite development has occurred, and major organs (including heart, kidney, gastrointestinal tract, thyroid gland, and thymus) and limb buds have developed.[20,21] The low-dose group (group I) received the adult human dose (10 mg/kg) throughout the major period of embryogenesis (days 6–11), while the medium-dose groups (groups II and III) received roughly 3 times the adult human dosage (30 mg/

kg). The medium-dose groups were comprised of group II, which received the dose early in embryogenesis (days 6–8) and group III, which received the dose later during the period of embryonic development (days 9–11). Group IV, the high-dose group (150 mg/kg/day), receiving 15 times the usual human therapeutic dose, was treated on days 6–11. The purpose of the high-dose group was to magnify any teratogenic effect, and the medium-dose groups were intended to place any effect temporally within the period of embryogenesis.

The animals were sacrificed by decapitation on days 18–19 of pregnancy (mean, 18.6, SD = 0.5) and the fetuses were immediately harvested by hysterectomy. The fetuses were then fixed in 10% formalin for several weeks. The uteri were examined immediately for macroscopic evidence of early pregnancy resorption, and the implantation sites were counted. Pregnancy resorptions were classified as implantation sites with no products of conception being recovered. Early intrauterine deaths were defined as the recovery of products of conception in a macerated state and the absence of eye pigmentation. Eye pigmentation is visible by the 14th day postconception,[22] so late intrauterine deaths were defined as dead fetuses with eye pigmentation. None of the recovered dead fetuses had eye pigment present.

The fetuses were examined under both low (15X) and high (45X) power microscopy by two trained observers. They were examined externally for visible morphologic defects and dissected for gross examination of defects, as described elsewhere.[22] Particular attention was paid to the thymus, heart and great vessels, genitourinary organs, adrenal glands, hard and soft palate, and intracerebral ventricles. The examiners were blinded to the drug exposure status of the fetuses, i.e., zidovudine-exposed vs. controls, through a numbered encoding system.

The statistical analysis was performed using either chi-squared analysis for comparison between groups or Fisher exact test, where appropriate. The analysis of variance was used to compare maternal weight and the number of offspring between the control and treatment groups. The Newman-Keuls multiple-group comparison test was used to establish which groups were significantly different. The litter effect was controlled for by an analysis of covariance, and it was not significant ($P > 0.05$).

### RESULTS

Nonpregnant animals were excluded from the study. There were 15 pregnancies in groups I and III, 13 in group II, 17 in group IV, and 14 in group V (control). There was no significant difference in maternal weight gain during pregnancy among the five treatment groups, although the difference between groups IV and V approached significance ($P < 0.09$). The groups were compared with regard to litter size (Table 1). The mean litter size in group IV was 6.7 compared with 11.4 in the control group ($P < 0.01$).

All uteri were examined for macroscopic evidence of early pregnancy resorptions. There was one pregnancy resorption in group I. There were 6 resorptions in group II, 5 in group III, 46 in group IV, and 2 in group V (control). The difference between group II and group V (control) approached statistical significance. The frequency of pregnancy resorptions in group IV was significantly higher ($P < 0.01$) than in any of the groups studied. Autopsies were performed on all 639 fetuses (Table 1). The frequency of congenital anomalies was not different among the groups ($P = 0.9$). The frequency of congenital anomalies was near to the expected for the animal species used in this investigation (1%). The power calculation indicated that an 8-fold increase in the frequency of congenital anomalies would have been detected in the present investigation at 80% power. The power of the current investigation to detect a 2-fold increase in the frequency of congenital anomalies was 40% with $\alpha = 0.05$.

### DISCUSSION

Zidovudine was not associated with an increased frequency of congenital anomalies in Sprague-Dawley rats at the doses give. Group IV (receiving 150 mg/kg/day during embryogenesis) had a greater number of resorptions than any of the other groups ($P < 0.01$). This finding suggests that the medication may be embryocidal. The embryolethal effect observed in this study of postimplantation rat embryos is consistent with the deleterious effect previously demonstrated in preimplantation mouse embryos.[15–18]

It is conceivable that the apparent embryocidal effect may not have been from embryotoxicity per se. The agent may have caused such severe early malformations of major organ systems that the em-

**TABLE 1. Pregnancy outcome in zidovudine-treated and control rats[a]**

| Group no. and treatment | Litters (no.) | Implantation sites (no.) | Fetuses No. | M | SE | Resorptions[b] No. | (%) | Intrauterine deaths[c] No. | (%) | Anomalies No. | (%) |
|---|---|---|---|---|---|---|---|---|---|---|---|
| I. Low dose early and late (10 mg/kg)[d] | 15 | 159 | 158 | 10.6 | 1.2 | 1 | (0.6) | 0 | — | 0 | — |
| II. Medium dose early (30 mg/kg)[e] | 13 | 137 | 120 | 9.3 | 1.4 | 6 | (5.0) | 11 | (9.2) | 1[f] | (0.8) |
| III. Medium dose late (30 mg/kg)[d] | 15 | 147 | 138 | 9.3 | 1.2 | 5 | (3.6) | 4 | (2.9) | 2[f] | (1.5) |
| IV. High dose early and late (150 mg.kg)[g] | 17 | 167 | 113 | 6.7 | 1.5 | 46 | (40.7) | 8 | (7.1) | — | — |
| V. Control[h] | 14 | 162 | 160 | 11.4 | 1.1 | 2 | (1.3) | 0 | 0 | 1[i] | (0.6) |
| Totals | 74 | 772 | 689 | 9.7 | 1.3 | 60 | (7.8)[b] | 23 | (3.0)[b] | 4 | (0.6)[c] |

[a]Nonpregnant animals excluded from this analysis; 18 animals were initially assigned to each group, and number of litters reflects only pregnant animals. The footnotes explain these exclusions by group.
[b]Denominator is number of implantation sites.
[c]Denominator is number of fetuses.
[d]Three were not pregnant, or preimplantation loss.
[e]Five were not pregnant, or preimplantation loss.
[f]Anencephaly.
[g]One was not pregnant, or preimplantation loss.
[h]Four were not pregnant, or preimplantation loss.
[i]Bilateral absence of digits of front limbs.

bryo died and was subsequently resorbed, which might explain the lack of teratogenic effect noted in initial reproductive toxicology studies when the drug was given to pregnant rats at doses up to 20 times the recommended human dose.[11] It is also possible that no teratogenic effect was seen in this study because once-daily dosing acutely exposed the fetuses to relatively small amounts of zidovudine. However, in a study from our laboratory using pregnant Long-Evans rats, the agent was concentrated in fetal and placental tissues when administered as a single oral dose of 30 mg/kg/day. Fetal and placental zidovudine levels remained elevated 6 h after maternal administration despite a rapid clearance from the maternal circulation.[12]

Caution must be used in the extrapolation of findings from animal teratology studies and their application to the human situation. Of more than 2,000 drugs that have been tested in animal models, approximately 55% were associated with congenital anomalies at high doses. Conversely, there are only 30–40 known human teratogens.[23,24] Importantly, the sensitivity of animal models for teratology studies is not 100%. Thalidomide, one of the most highly publicized and tragic human teratogens, shows species-specific teratogenesis and is only a very weak teratogen in rats.[25]

The teratogenicity of zidovudine in humans is an important issue. Twenty to fifty percent of the infants of HIV-infected women will contract the uniformly fatal disease AIDS.[4] Zidovudine has the ability to decrease the viral antigenemia in vivo and to inhibit replication and infectivity of the virus in vitro; it also has a profound effect on the frequency and severity of neonatal infection.[4,26,27] Zidovudine has no proven teratogenic activity in rats. However, reliable statements about human teratogenicity of zidovudine must await studies of the offspring of nonhuman primates or humans who were exposed to the drug during early prenatal development. These results strongly suggest that zidovudine may be detrimental to early embryonic development, possibly related to inhibited cell hyperplasia. Embryofetal lethality is an indicated risk of zidovudine therapy during early pregnancy.

### REFERENCES

1. Fischl MA, Richman DD, Grieco MH, et al.: The efficacy of azidothymidine (AZT) in the treatment of patients with AIDS and AIDS related complex. N Engl J Med 317:185–191, 1987.
2. Yarchoan R, Berg G, Browers P, et al.: Response of human-immunodeficiency-virus associated neurologic disease to 3'-azido-3'deoxythymidine. Lancet 1:132–135, 1987.

3. Jackson GG, Paul DA, Falk LA, et al.: Human immunodeficiency virus (HIV) antigenemia in the acquired immunodeficiency virus syndrome (AIDS) and the effect of treatment with zidovudine (AZT). Ann Intern Med 108: 175–180, 1988.
4. Centers for Disease Control: Zidovudine therapy for HIV in pregnancy. MMWR 43:285–287, 1994.
5. Burke DS, Brundage JF, Herbold JR, et al.: Human immunodeficiency virus infections among civilian applicants for United States military service, October 1985 to March 1986: Demographic factors associated with seropositivity. N Engl J Med 317:131–136, 1987.
6. Landesman S, Minkoff H, Holman S, McCalla S, Sijin O: Serosurvey of human immunodeficiency virus infection in parturients: Implications for human immunodeficiency virus testing programs of pregnant women. JAMA 258:2701–2703, 1987.
7. Centers for Disease Control: Human immunodeficiency virus infection in the United States: A review of current knowledge. MMWR 35(suppl 6):1–20, 1987.
8. Furman PH, Fyle JA, St Clair MH, et al.: Phosphorylation of 3′azido-3′-deoxythymidine and selective interaction of the 5′triphosphate with human immunodeficiency virus reverse transcriptase. Proc Natl Acad Sci USA 83: 8333–8337, 1986.
9. Mitsuya H, Weinhold KJ, Furman PA, et al.: 3′Azido-3′-deoxythymidine (BW A509U): An antiviral agent that inhibits the infectivity and cytopathic effect of human T-lymphotropic virus type III/lympadenopathy associated virus in vitro. Proc Natl Acad Sci USA 82:7096–7100, 1985.
10. Richman DD, Fischl MA, Grieco MH, et al.: The toxicity of azidothymidine (AZT) in the treatment of AIDS and AIDS-related complex. A double blind placebo-controlled trial. N Engl J Med 317:192–197, 1987.
11. Physician's Desk Reference, 42nd ed. Oradell, NJ: Medical Economics Co., p 820, 1988.
12. Little BB, Bawdon RA, Christmas JT, Sobhi S, Gilstrap LC: Pharmacokinetics of zidovudine during late pregnancy in Long Evans rats. Am J Obstet Gynecol 161: 732–734, 1989.
13. Liebes L, Mendoza S, Wilson D, Dancis J: Transfer of zidovudine (AZT) by human placenta. J Infect Dis 161: 203–207, 1990.
14. Bawdon RE, Sobhi S, Dax J: The transfer of anti-HIV nucleoside compounds by the term human placenta. Am J Obstet Gynecol 167:1570–1574, 1992.
15. Klug S, Lewandowski C, Merker HJ, Stahlmann R, Wildi L, Neubert D: In vitro and in vivo studies on the prenatal toxicity of five virustatic nucleoside analogues in comparison to aciclovir. Arch Toxicol 65:283–291, 1991.
16. Gogu SR, Beckman BS, Agrawal KC: Amelioration of zidovudine-induced fetal toxicity in pregnant mice. Antimicrob Agents Chemother 36:2370–2374, 1992.
17. Sieh E, Coluzzi ML, Cusella DeAngelis MG, et al.: The effects of AZT and DDI on pre- and postimplantation mammalian embryos: An in vivo and in vitro study. AIDS Res Hum Retroviruses 8:639–649, 1992.
18. Toltzis P, Mourton T, Magnuson T: Effect of zidovudine on preimplantation murine embryos. Antimicrob Agents Chemother 37:1610–1613, 1993.
19. Palmer AK: The design of subprimate animal studies. In Wison JG, Fraser FC (eds): Handbook of Teratology. Vol 4, Research Procedures and Data Analysis. New York: Plenum Press, pp 215–253, 1978.
20. Nicholas JS: Experimental methods and rat embryos. In Farris EJ, Griffith JQ (eds): The Rat in Laboratory Investigation. New York: Hafner Publishing, pp 51–67, 1962.
21. Witschi E: Development of rat. In Altman P, Dittmer DS (eds): Growth Including Reproduction and Morphological Development. Washington, DC: Fed Amer for Exp Biol, pp 304–414, 1962.
22. Taylor P: Practical Teratology. London: Academic Press, Harcourt-Brace-Jovanovich, 1986.
23. Shepard TH: Catalog of Teratogenic Agents. Baltimore: Johns Hopkins University Press, 1989.
24. Schardein J: Chemically Induced Birth Defects. 2nd ed. New York: Dekker. 1993.
25. Shumacher H, Blake DA, Gurian JM, Gillette JR: A comparison of the teratogenic activity of thalidomide in rabbits and rats. J Pharmacol Exp Ther 160:189–200, 1968.
26. Viscarello RR, DeGennaro NJ, Hobbins JC: Preliminary experience with the use of zidovudine (AZT) during pregnancy. Am J Obstet Gynecol 164:248, 1991.
27. Chavanet P, Diquet B, Waldner A, Portier H: Perinatal pharmacokinetics of zidovudine. N Engl J Med 321: 1548–1549, 1989.
